# Cerebral oxygenation and autoregulation during rewarming on cardiopulmonary bypass

**DOI:** 10.1177/02676591211064961

**Published:** 2022-01-17

**Authors:** Henrik Arthursson, Gunilla Kjellberg, Thomas Tovedal, Fredrik Lennmyr

**Affiliations:** Department of Thoracic Surgery and Anesthesiology, 151670 Uppsala University Hospital, Uppsala, Sweden

**Keywords:** Cerebral autoregulation, rewarming, cardiopulmonary bypass, regional cerebral oxygenation, Cerebral Oximetry Index

## Abstract

**Background:**

Rewarming on cardiopulmonary bypass (CPB) is associated with increased metabolic demands; however, it remains unclear whether cerebral autoregulation is affected during this phase. This RCT aims to describe the effects of 20% supranormal, compared to normal CPB flow, on monitoring signs of inadequate perfusion, oxygenation, and disturbed cerebral autoregulation, during the rewarming phase of CPB.

**Method:**

Thirty two patients scheduled for coronary artery bypass grafting were allocated to a Control group (*n* = 16) receiving a CPB pump flow corresponding to preoperatively measured cardiac output, and an Intervention group (*n* = 16) receiving the corresponding CPB pump flow increased by 20% during rewarming. Cerebral Oximetry Index (COx) was calculated with the aid of Near Infrared Spectroscopy.

**Results:**

Twenty five patients were included in the data. Results show a median COx value of 0.0 (IQR −0.33–0.5) (Control) and 0.0 (IQR −0.15–0.25) (Intervention), respectively; *p* = .85 with individual variations within groups. The median cerebral perfusion pressure (CPP) was 55 (52–58) (Control) and 61 (54–66) mmHg (Intervention); *p* = .08. No significant difference in rSO2 values was observed between the groups (58.5% (50–61) versus 64% (58–68); *p* = .06).

**Conclusion:**

The present study showed no difference between increased and normal CPB pump flow with respect to cerebral autoregulation during rewarming. Large variations in cerebral autoregulation were seen at individual level.

## Introduction

Open cardiac surgery is typically performed with the aid of hypothermia during cardiopulmonary bypass (CPB). The degree of hypothermia varies with the complexity and duration of the surgery, and comprises rewarming, during which the metabolic demands for blood flow and oxygen delivery are increased.^1^ The brain is especially sensitive to insufficient nutritive supply, due to its low capacity for anaerobic metabolism and its limited glycogen reserves.^2,3^

Adverse neurological effects due to CPB occur with an incidence between 1% and 6% for major neurology,^4,5^ while the incidence of neurocognitive deficits is reportedly much higher, up to 5%–40%.^2,6^ The cause is considered multifactorial, including events as hypoperfusion, embolism, and/or hypoxemia.^7^ Underlying co-morbidities such as hypertension, atherosclerosis, senility, and diabetes may all contribute, and are present in more than 50% of cardiac surgery patients.^2,8^ In hypothermic CPB, the restoring of body temperature itself may impose an increased neurological risk, but the exact mechanism has not been established.^3,9–11^ One of the physiological aspects of rewarming is the cerebral autoregulation (CA), and there are indications of an association between rewarming, impaired autoregulation, and possibly stroke.^9^

### Cerebral autoregulation

Cerebral autoregulation can be defined as the ability of the brain to maintain adequate perfusion across various mean arterial blood pressure (MAP). A positive correlation between MAP and cerebral perfusion parameters, with an *r* value above 0.3–0.5,^9,10,12^ is considered indicative of impaired autoregulation. By monitoring cerebral regional oxygen saturation (rSO2), a correlation value (Cerebral Oximetry Index, (COx)) may thus be obtained.^13,14^ COx has previously been validated as a surrogate for the transcranial Doppler (TCD)-derived parameter Mean Velocity Index (Mx).^10,11,13^ Cerebral blood flow can be estimated by measuring the blood velocity in the middle cerebral artery (MCA) using TCD-technique.^15^ Although, the method is limited by lack of temporal sonic window in 10–20% of patients.^15,16^

Impaired autoregulation would render the brain more susceptible to hypoperfusion related to low cerebral blood flow, which in turn depends on the CPB flow. This study aims to describe the effects of 20% supranormal CPB blood flow, compared to normal CPB blood flow, on monitoring signs of inadequate perfusion, oxygenation, and disturbed CA during rewarming.

The null hypothesis was that the groups were similar in CA parameters during rewarming.

## Materials and Method

This blinded RCT was conducted at the Department of Cardiothoracic Surgery and Anesthesia, Uppsala University Hospital, Sweden. Ethical approval for this study was granted by the Swedish Ethical Review Authority (Dnr 2017/275) prior to initiation.

### Study population

The study included 32 adult patients scheduled for coronary artery bypass grafting (CABG). The patients were randomized (closed envelope) to either a group with a CPB pump flow based on individual cardiac output (CO) as determined by thermodilution prior to CPB (Control group, *n* = 16) or a CPB pump flow based on individual CO + 20% (Intervention group, *n* = 16).

Exclusion criteria were carotid artery stenosis, previous cerebral vascular insult, known pathological intracerebral processes, atherosclerosis of the ascending aorta, a left ventricular ejection fraction (LVEF) <0.30, measured CI prior to CPB <2.1 L/min/m^2^, and/or extended surgery due to unexpected findings or events.

### Brain oxygenation and cerebral blood flow

Brain oxygenation was measured using near infrared light spectroscopy (NIRS), which is an established non-invasive technique.^17^ The optodes (INVOS™, Medtronic Inc., Minneapolis, MN) were placed bilaterally on the forehead prior to preoxygenation and anesthesia induction, and rSO_2_ was monitored continuously throughout the surgery.

Using measured rSO_2_ and cerebral perfusion pressure (CPP) (defined as the difference between MAP and central venous pressure (CVP)),^18^ assuming that intracranial pressure was lower than CVP,^19–22^ COx was calculated using the Spearman rank correlation test.^12,13^

Values for CPP and rSO_2_ were collected every minute, and individual COx value for the patients of each group was calculated. In total, three COx values were calculated during rewarming, between 32°C–34°C, 34.1°C–35.5°C, and 35.6°C–37°C.

### Perioperative monitoring and anesthesia

Left radial artery blood pressure monitoring and blood gas sampling were used, and the anesthesia consisted of propofol and fentanyl, combined with rocuronium muscle relaxation. After intubation, anesthesia was maintained by sevoflurane, fentanyl/alfentanil, and isoflurane during CPB. Temperatures were measured in the nasopharynx.

CO was measured using thermodilution with a pulmonary artery catheter (Swan-Ganz catheter model 132F5, Edwards Lifesciences, Irvine CA) just after insertion with stable circulatory condition, normal body temperature, hematocrit above 30%, and pulse rate at a minimum of 50 strokes/min. CO was measured at a minimum of three occasions, using mean value.

CPB was performed with a Stöckert S5 roller pump (Livanova, London, UK) heart–lung machine with a custom-made PVC tubing set produced by Maquet (Getinge Group, Gothenburg, Sweden). In-line monitoring consisted of B-Care5 (Livanova) and a CDI® Blood Parameter Monitoring System 500 (Terumo, Somerset, UK). Once on CPB, patients were cooled to 32°C.

In group 1 (control), the pump flow equaled the individual CO as measured prior to CPB, while in group 2 (intervention), the pump flow was set to 120% of the individual CO, with this flow initiated at the time point of rewarming. The flow rate was maintained during rewarming without adjustments for temperature during hypothermia. The PaCO_2_ was kept as close as possible to a goal of 5.0 kPa using alpha stat monitoring. The PaO_2_ was kept at normal levels at the perfusionists’ discretion, while the lower span for hematocrit, SvO_2_, and MAP were >24%, >70%, and >55 mmHg, respectively. The MAP was maintained using norepinephrine as a vasopressor, with doses ranging between zero and 0.2 μg/kg/min.

For rewarming, the heater–cooler was set to 37.5–38°C, and with oxygenator arterial outlet temperature <37.5°C.

Hemodynamics and rSO_2_ data registration were performed every minute during rewarming.

### Statistics

Normality tests were performed with the Shapiro–Wilks normality test. As a Gaussian sample data distribution could not be assumed, between-group comparisons were made with the non-parametric Mann–Whitney test, and correlations were analyzed with the Spearman rank test. All data are presented as medians and interquartile range (IQR). Statistical analysis was performed with GraphPad Prism 8/9 (GraphPad Software Inc. La Jolla, CA) and with Microsoft Excel (Microsoft Corporation, Redmond, WA). An alpha level of 5% (*p* < .05) was considered statistically significant.

The statistical power was estimated to be 60% based on the mean rSO_2_ values of 59% and 64%, respectively, an SD of 5.9% and alpha 0.05, with results from the present study.

## Results

**Figure 1.**
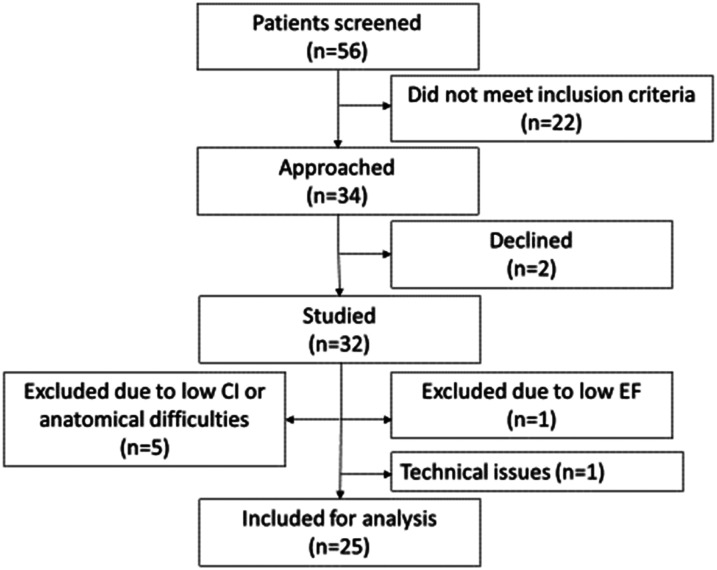
Flow diagram of the patients enrolled in the study and included for analysis. CI: Cardiac Index; EF: Ejection Fraction.

Fifty-six patients were screened of which 34 were found eligible and eventually 32 accepted to participate. Of those 32 patients, seven patients were excluded due to low perioperatively discovered LVEF (*n* = 1), low CI prior to CPB (*n* = 5), and due to anatomical and technical reasons (*n* = 1) (Figure 1).

### Demographics

At baseline, there was a difference in CO between the groups (4.2 vs 4.8 L/min; control vs intervention; *p* = .05); however, the adjusted CI was similar between groups (2.2 vs 2.3 L/min/m^2^; *p* = .18). CPB blood flow between groups showed a statistically significant difference (4.8 vs 5.0 L/min, *p* = .02). There was a statistically significant difference in aortic cross clamp time that was unrelated to the study protocol (52 min vs. 71 min; control vs. intervention group; *p* = .04). The demographics are described in Table 1, and the physiological data are described in Table 2.

**Table 1.** Demographics and perioperative data. Medians and IQR.

	Control group (*n* = 11)	Intervention group (*n* = 14)	*p*-value
Age (years)	69 (65–71)	73 (60–74)	.28
Weight (kg)	93 (72–102)	83 (74–92)	.28
Length (cm)	174 (170–178)	174 (166–178)	.58
BSA (m^2^)	2.07 (1.89–2.19)	2.0 (1.84–2.07)	.20
Cardiac output (L/min)	4.8 (4.2–4.9)	4.2 (4.0–4.6)	.03
Cardiac index	2.3 (2.2–2.4)	2.2 (2.1–2.3)	.18
Rewarming time	30 (25–36)	33 (26–37)	.56
Cardiopulmonary bypass blood flow (L/min)	4.8 (4.2–4.9)	5.0 (4.8–5.5)	.02
Time surgery (min)	89 (83–124)	102 (89–113)	.44
Time aortic clamp (min)	52 (43–70)	71 (59–73)	.04
Prior myocardial infarction	5 (46%)	4 (29%)	.42
Hypertension	7 (64%)	4 (29%)	.12
Diabetes	3 (27%)	5 (36%)	.69

BSA: Body Surface Area.

**Table 2.** Rewarming physiological measurements. Medians and IQR.

	Control group (*n* = 11)	Intervention group (*n* = 14)	*p*-value
PaCO_2_ (kPa)	5.1 (5.0–5.2)	5.0 (4.8–5.1)	.10
PaO_2_ (kPa)	21.3 (20.2–23.6)	21.0 (19.4–21.9)	.37
Hematocrit (%)	33 (30–34)	31 (27–35)	.72
Central venous pressure (mmHg)	6 (3–7)	4 (1–7)	.34
SvO_2_ (%)	73 (71–79)	77 (75–80)	.06
Mean arterial blood pressure (mmHg)	61 (58–62)	65 (60–69)	.06

CVP: Central Venous Pressure; MAP: Mean Arterial Pressure.

### COx values and cerebral oximetry

**Figure 2.**
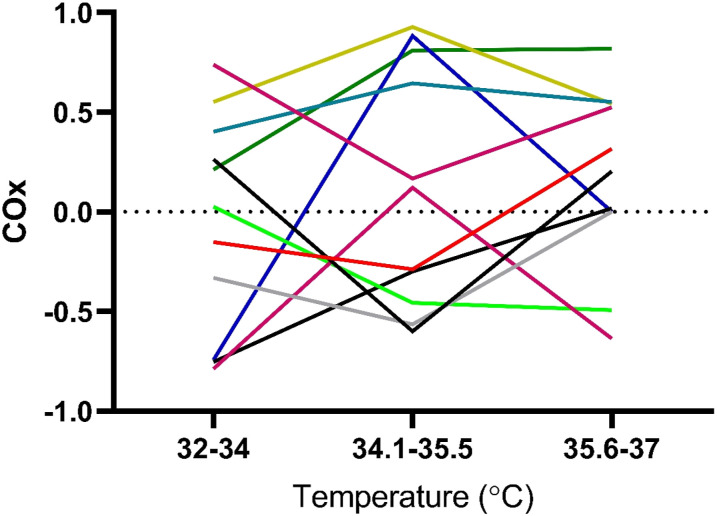
Individual COx (Cerebral Oximetry Index) values in Control group (n=11) deriving from in between different correlation points. The result show large variations on individual level, although no statistically significant difference compared to the Intervention group.

**Figure 3.**
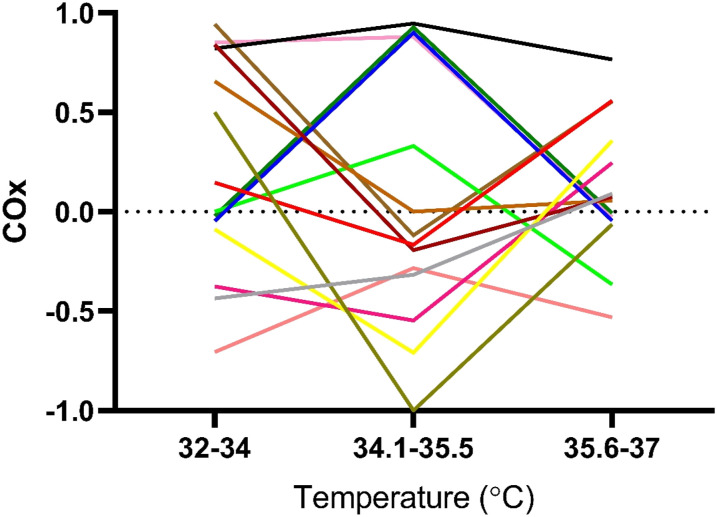
Individual COx (Cerebral Oximetry Index) values in Intervention group (n=14) deriving from in between different correlation points. The result show large variations on individual level, although no statistically significant difference compared to Control group.

A wide variation of COx values was seen in both groups, and there was no consistent trend across the rewarming in neither group nor no significant difference between the groups. The COx value as calculated for each of the three periods of rewarming (32–34°C; 34.1–35.5°C; 35.6–37°C) between groups (Table 3) and results are displayed per individual and group (Figures 2 and 3).

**Table 3.** Rewarming COx (Cerebral Oximetry Index) r values at different measuring points in groups respectively. Medians and IQR.

Temperature (°C)	Control group (*n* = 11)	Intervention group (*n* = 14)	*p*-value
32–34	0.03 (−0.74–0.4)	0.07 (−0.16–0.83)	.27
34.1–35.5	0.12 (−0.46–0.81)	−0.14 (−0.37–0.89)	.99
35.6–37	0.20 (0.0–0.54)	0.07 (−0.05–0.41)	.69

There was no significant difference in CPP between the groups (55 (52–58) mmHg vs 61 (54–66) mmHg; control versus intervention; *p* = 0.08). In both groups, there was a decrease in CPP over the rewarming period and no significant difference could be seen between the groups (−19% vs −24%; control vs intervention; *p* = 0.44) (Supplementary Figure 4).

No significant differences in rSO_2_ values were observed between the groups (59 (50–61) % vs 64(58–68) %; control versus intervention; *p* = .06) (Supplementary Figure 5).

## Discussion

The main finding of the present study is that increasing the pump flow rate by 20% above the individual baseline level did not significantly alter the monitored parameters of cerebral autoregulation during rewarming.

In this study, the CPB pump flow was tailored using the CI after induction of anesthesia as a perfusion benchmark, the purpose of which was to reduce the inter-individual variations due to discrepancies between the actual CI and the applied perfusion index. In order to minimize the risk of hypoperfusion using this method, patients with low LVEF <0.30 were excluded and no perfusion index was set lower than 2.1 L/min/m^2^. This is a lower perfusion index than the usually used 2.2–2.4 index,^2,23^ although indexes with lower limits have been used in several studies^3,24,25^ without jeopardizing patient safety. In the light if this and recommendations from EACTS/EACTA/EBCP,^23^ where the use of BSA has been discussed and Lean Body Mass (LBM) has been suggested as a more sensitive measurement of metabolic needs regarding CPB flow, the lower indexes used in this study could be considered safe. Aortic cross clamp time differed significantly between groups due to surgical factors.^26^ However, this difference was regarded as unlikely to affect the results, since the measurements focused on the rewarming phase, that is, after the release of the aortic cross clamp.

Cardiopulmonary bypass blood flow showed a statistically significant difference between groups (*p* = .02) with an increase of blood flow by 20%. It appears that this 20% increase did not significantly affect the COx values, but interestingly, there was a tendency toward higher venous saturations, cerebral perfusion pressure, and cerebral oxygenation in the intervention group compared with controls. All values were within the normal range; thus, leaving no clear indication on what approach, if any, would be superior to the other.

While low perfusion pressure and venous desaturation are classical warning signs of hypoperfusion, it has also been pointed out that excess cerebral blood flow and pressure do carry risks of barotrauma and increased embolic load that may add to the risk for cerebral damage.^27^ It has been demonstrated that a slower rate of rewarming, with lower CPB perfusate temperature gradients, may improve cognitive performance after cardiac surgery.^3^ Moreover, excessive rewarming may increase the risk of acute kidney injury,^28^ which suggests that rewarming should be performed with caution; however, questions remain regarding the optimal blood flow. In the present study, rewarming rate lies well below the recommended <0.5°C/min,^29^ and comparing rewarming times between groups showed no statistically significant difference in this study (*p* = .56).

The cerebral perfusion pressure is a vital circulatory parameter. In the present study, the MAP was kept >55 mmHg with the aid of vasopressor, but importantly, the primary intervention was directed toward blood flow rather than arterial pressure. It should be noted that in previous studies where CA was preserved, the MAP level was higher than in the present study.^30^ It is also true that large variations exist between patients and that the difficulties in predicting the effects on CA may suggest a need for specific perioperative monitoring, for example, with COx.^13^ While the present study excluded patients with neurological risk factors, the study by Hori et al.^30^ did target patients with a high risk of neurological complications, and also applied a higher MAP than in the present material. Regardless of the neurological risk, flow and pressure aspects co-exist in each patient, and systematic monitoring is likely to be required in the clinical setting to understand the effects on CA.

At group level, the COx values do not suggest that CA would be affected by the choice of CPB pump flow level in neither group. However, the variations within the groups were marked and it cannot be excluded that CA was affected in individual cases. Notably, the present material covered only the rewarming phase, but in other studies, CA was analyzed over the entire CPB span and then showed that CA could be affected.^12,18,25,26^ There is also specific data from rewarming in other materials, where CA was affected after mild hypothermia^9^ and after deep hypothermia.^10^ Interestingly, it has been showed that CA may be impaired in a minority of the patients subjected to CPB and that this may predispose to postoperative neurological complications.^11^ This would be in line with the observations of variation in the present material and supports the role of monitoring in order to enable prevention of harmful events.

Regional cerebral oximetry saturation (rSO_2_) measurements showed no significant difference between the examined groups. In general, rSO_2_ values show large variations between the individual patients. Changes in NIRS such as a uni- or bilateral reduction from the baseline value by 20% or an absolute decrease by 50% have been described as pathological.^17,28,29^ The usefulness of, and verification of, rSO_2_ values in clinical practice has been previously shown,^10,13,14,17,28–31^ and general, following trends may be more informative than comparing absolute values between patients.^32^

### Limitations of the study

Despite being the gold standard for measuring CO,^33^ the accuracy of the thermodilution method relies on stabile conditions including the physiology and repeated measurements can indeed show some variation. The use of triple measurements is believed to reduce the variations sufficiently.

An unexpected difference regarding cross clamp time between groups (52 vs 71 min, *p* = .04) was noted, which could have resulted in lower core temperature in the intervention group, and it cannot be ruled out that this has affected other perfusion parameters during CPB.

The present study was performed on a small sample size (*n* = 25) with CABG patients only, which limits the interpretation of the results and implies an increased risk for false-negative results.

The exclusion criteria were carotid artery stenosis, previous cerebral vascular insult, and known pathological intracerebral processes. Patients with such pathological processes may be suspected of being more sensitive to changes in CA, and possibly, such patients may benefit more than average from using a higher perfusion index and MAP to avoid neurological complications. Research in this area should be prioritized since many of the cardiac surgery patients belong to this fragile group, as was addressed by Hori et al.^30^ in 2017.

## Conclusion

The result of this study shows no apparent difference in impact on cerebral autoregulation during rewarming by a CPB pump flow equal to the individual CO as compared with 120% of the same pump flow. However, individual responses compatible with affected CA could not be ruled out.

## Supplementary Material

Supplementary material
