# Supplementary figures and images for: Cerebral oxygenation and autoregulation during rewarming on cardiopulmonary bypass
Source: Perfusion. 2022 Jan 17;38(3):523–9. doi: 10.1177/02676591211064961 (PMC10026164; doi:10.1177/02676591211064961)

Supplementary Figures

Supplementary figure 4.

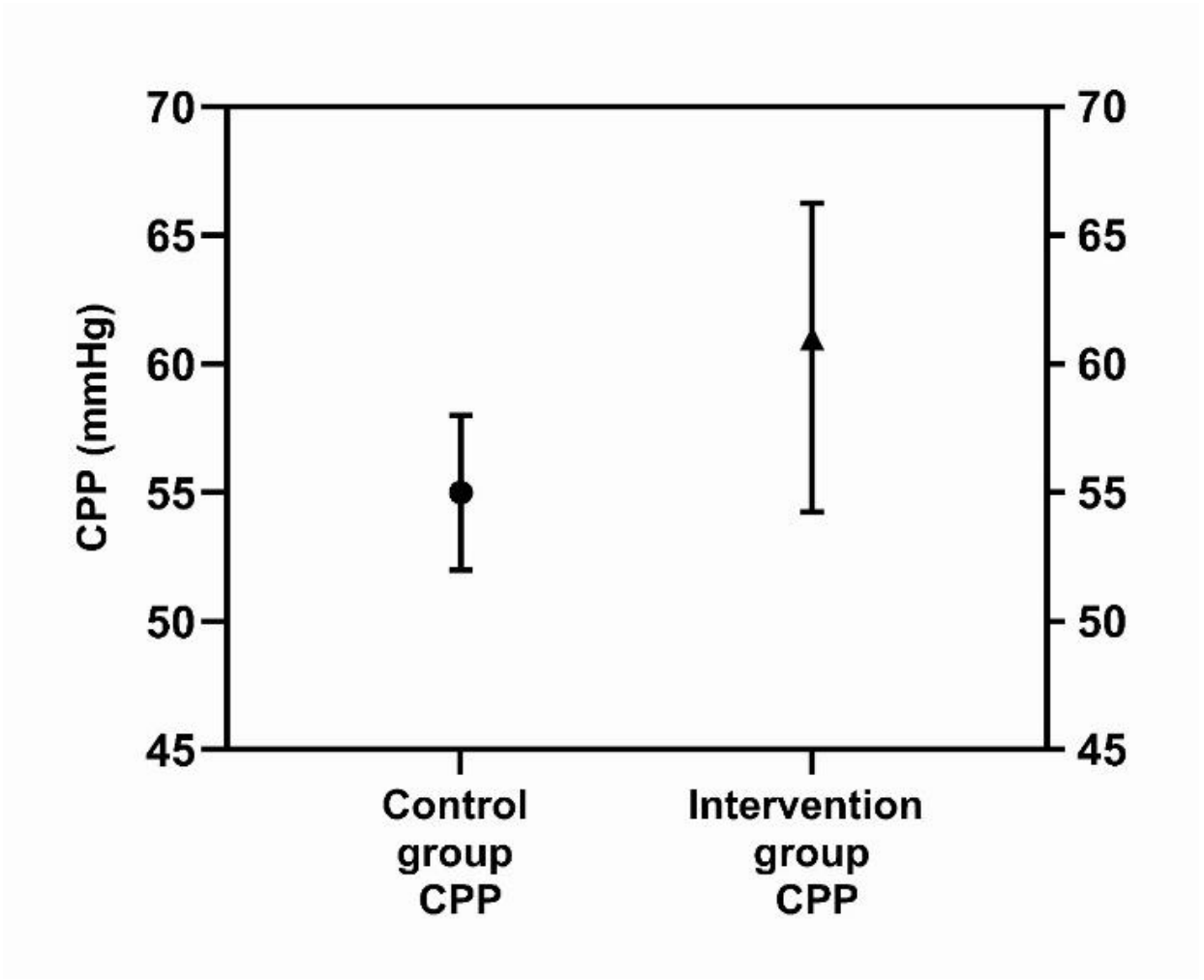

Supplementary figure 5.

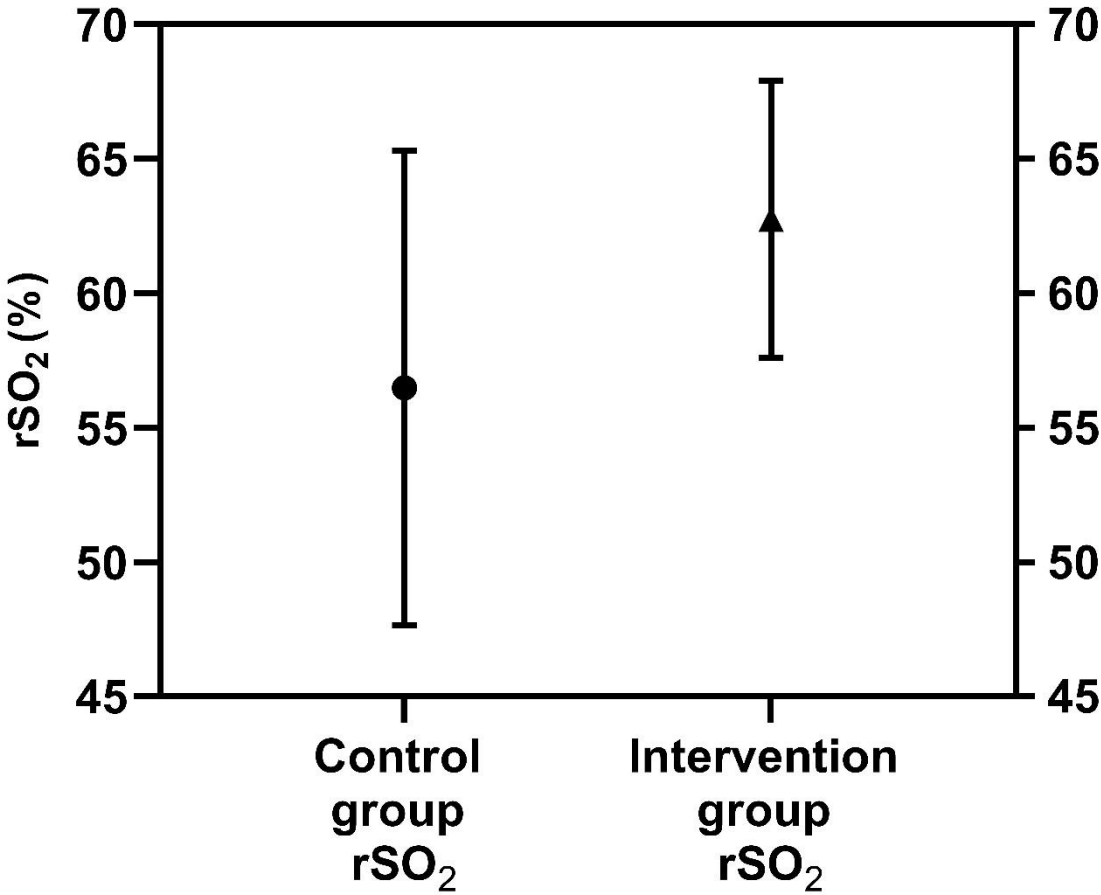

Supplement: Supplementary material [file sj-pdf-1-prf-10.1177_02676591211064961.pdf]
